# Supplementary material for: Neoadjuvant sintilimab in combination with concurrent chemoradiotherapy for locally advanced gastric or gastroesophageal junction adenocarcinoma: a single-arm phase 2 trial
Source: Nat Commun. 2023 Aug 14;14:4904. doi: 10.1038/s41467-023-40480-x (PMC10425436; doi:10.1038/s41467-023-40480-x)
Supplement: Supplementary file 1 — Supplementary Information [file 41467_2023_40480_MOESM1_ESM.pdf]

Supplementary Table 1. Concurrent chemoradiotherapy (cCRT)-related adverse events.

| Event, n (%) | Grade 1 | Grade 2 | Grade 3 | Grade 4 | Any Grade |
|---|---|---|---|---|---|
| Myelosuppression | 3 (8.8) | 10 (29.4) | 7 (20.6) | 4 (11.8) | 24 (70.6) |
| Nausea/vomiting | 7 (20.6) | 6 (17.6) | 4 (11.8) | 0 (0.0) | 17 (50) |
| Fever | 2 (5.9) | 0 (0.0) | 1 (2.9) | 0 (0.0) | 3 (8.8) |
| Fatigue | 1 (2.9) | 1 (2.9) | 0 (0.0) | 0 (0.0) | 2 (5.8) |
| Decreased albumin | 4 (11.8) | 0 (0.0) | 0 (0.0) | 0 (0.0) | 4 (11.8) |
| Elevated creatinine | 0 (0.0) | 1 (2.9) | 0 (0.0) | 0 (0.0) | 1 (2.9) |

Supplementary Table 2. Immunotherapy-related adverse events (irAE).

| Event, n (%) | Grade 1 | Grade 2 | Grade 3 | Grade 4 | Any Grade |
|---|---|---|---|---|---|
| Rash | 5 (14.7) | 1 (2.9) | 2 (5.9) | 0 | 8 (23.5) |
| Hypopituitarism | 0 | 1 (2.9) | 0 | 0 | 1 (2.9) |
| Hyperthyroidism | 0 | 1 (2.9) | 0 | 0 | 1 (2.9) |
| Pneumonitis | 0 | 1 (2.9) | 0 | 0 | 1 (2.9) |
| Elevated transaminases | 0 | 0 | 1 (2.9) | 0 | 1 (2.9) |
| Elevated bilirubin | 0 | 0 | 0 | 1 (2.9) | 1 (2.9) |

Supplementary Table 3. Surgery-related adverse events (srAE).

| Event, n (%) | Grade 1 | Grade 2 | Grade 3 | Grade 4 | Any Grade |
|---|---|---|---|---|---|
| Elevated transaminases | 5 (14.7) | 0 | 0 | 0 | 5 (14.7) |
| Decreased albumin | 3 (8.3) | 0 | 0 | 0 | 3 (8.3) |
| Hypokalemia | 5 (14.7) | 1 (2.9) | 0 | 0 | 6 (17.6) |
| Pneumonia | 0 | 1 (2.9) | 0 | 0 | 1 (2.9) |
| Anastomotic leakage | 0 | 1 (2.9) | 0 | 0 | 1 (2.9) |
| Intestinal obstruction | 0 | 0 | 1 (2.9) | 0 | 1 (2.9) |

Supplementary Table 4. Adjusted P-values of biomarker analysis

| Sampling time | Biomarker | P-value | Adjusted P-value |
|---|---|---|---|
| Baseline | CD3+ | 0.039 | 0.468 |
| | CD4+ | 0.277 | 1.000 |
| | CD56+ | 0.026 | 0.312 |
| | CD56 bright | 0.048 | 0.576 |
| | CD56 dim | 0.018 | 0.216 |
| | PD-1 | 0.487 | 1.000 |
| | FoxP3+ | 0.477 | 1.000 |
| | CD20+ | 0.369 | 1.000 |
| | CD8+ | 0.878 | 1.000 |
| | CD68+CD163- | 0.975 | 1.000 |
| | CD68+CD163+ | 0.393 | 1.000 |
| | CD68+CD163-/CD68+ | 0.276 | 1.000 |
| | CD3+(stroma) | 0.336 | 1.000 |
| | CD4+(stroma) | 0.018 | 0.216 |
| | CD56+(stroma) | 0.002 | 0.024 |
| | CD56 bright(stroma) | 0.013 | 0.156 |
| | CD56 dim(stroma) | 0.001 | 0.012 |
| | PD-1(stroma) | 0.769 | 1.000 |
| | FoxP3+(stroma) | 0.721 | 1.000 |
| | CD20+(stroma) | 0.369 | 1.000 |
| | CD8+(stroma) | 0.109 | 1.000 |
| | CD68+CD163-(stroma) | 0.547 | 1.000 |
| | CD68+CD163+(stroma) | 0.168 | 1.000 |
| | CD68+CD163-/CD68+(stroma) | 0.033 | 0.396 |
| Fold change from baseline to | CD20+ | 0.814 | 1.000 |

| | | | |
|---|---|---|---|
| posttreatment | | | |
| | CD3+ | 0.139 | 1.000 |
| | CD4+ | 0.139 | 1.000 |
| | CD8+ | 0.481 | 1.000 |
| | FoxP3+ | 0.815 | 1.000 |
| | CD56 | 0.888 | 1.000 |
| | CD56 bright | 0.887 | 1.000 |
| | CD56 dim | 0.888 | 1.000 |
| | PD-1 | 0.541 | 1.000 |
| | CD68+CD163- | 0.815 | 1.000 |
| | CD68+CD163+ | 0.541 | 1.000 |
| | CD68+CD163-/CD68+ | 0.277 | 1.000 |
| | CD20+(stroma) | 0.423 | 1.000 |
| | CD3+(stroma) | 0.236 | 1.000 |
| | CD4+(stroma) | 0.059 | 0.708 |
| | CD8+(stroma) | 0.370 | 1.000 |
| | FoxP3+(stroma) | 1.000 | 1.000 |
| | CD56(stroma) | 0.481 | 1.000 |
| | CD56 bright(stroma) | 0.714 | 1.000 |
| | CD56 dim(stroma) | 0.481 | 1.000 |
| | PD-1(stroma) | 0.423 | 1.000 |
| | CD68+CD163-(stroma) | 0.541 | 1.000 |
| | CD68+CD163+(stroma) | 0.134 | 1.000 |
| | CD68+CD163-/CD68+(stroma) | 0.423 | 1.000 |
| Posttreatment | CD20+ | 0.015 | 0.180 |
| | CD3+ | 0.123 | 1.000 |
| | CD4+ | 0.815 | 1.000 |
| | CD8+ | 0.861 | 1.000 |
| | FoxP3+ | 0.379 | 1.000 |

| | | |
|---|---|---|
| CD56 | 0.098 | 1.000 |
| CD56 bright | 0.912 | 1.000 |
| CD56 dim | 0.086 | 1.000 |
| PD-1 | 0.907 | 1.000 |
| CD68+CD163- | 0.640 | 1.000 |
| CD68+CD163+ | 0.318 | 1.000 |
| CD68+CD163-/CD68+ | 0.412 | 1.000 |
| CD20+(stroma) | 0.075 | 0.900 |
| CD3+(stroma) | 0.482 | 1.000 |
| CD4+(stroma) | 0.558 | 1.000 |
| CD8+(stroma) | 0.640 | 1.000 |
| FoxP3+(stroma) | 0.640 | 1.000 |
| CD56(stroma) | 0.142 | 1.000 |
| CD56 bright(stroma) | 0.865 | 1.000 |
| CD56 dim(stroma) | 0.126 | 1.000 |
| PD-1(stroma) | 0.907 | 1.000 |
| CD68+CD163-(stroma) | 0.682 | 1.000 |
| CD68+CD163+(stroma) | 0.340 | 1.000 |
| CD68+CD163-/CD68+(stroma) | 0.473 | 1.000 |

The comparison of immune cell infiltration levels was performed using Friedman's non-parametric test and the adjusted P-value were imputed using Bonferroni methods.

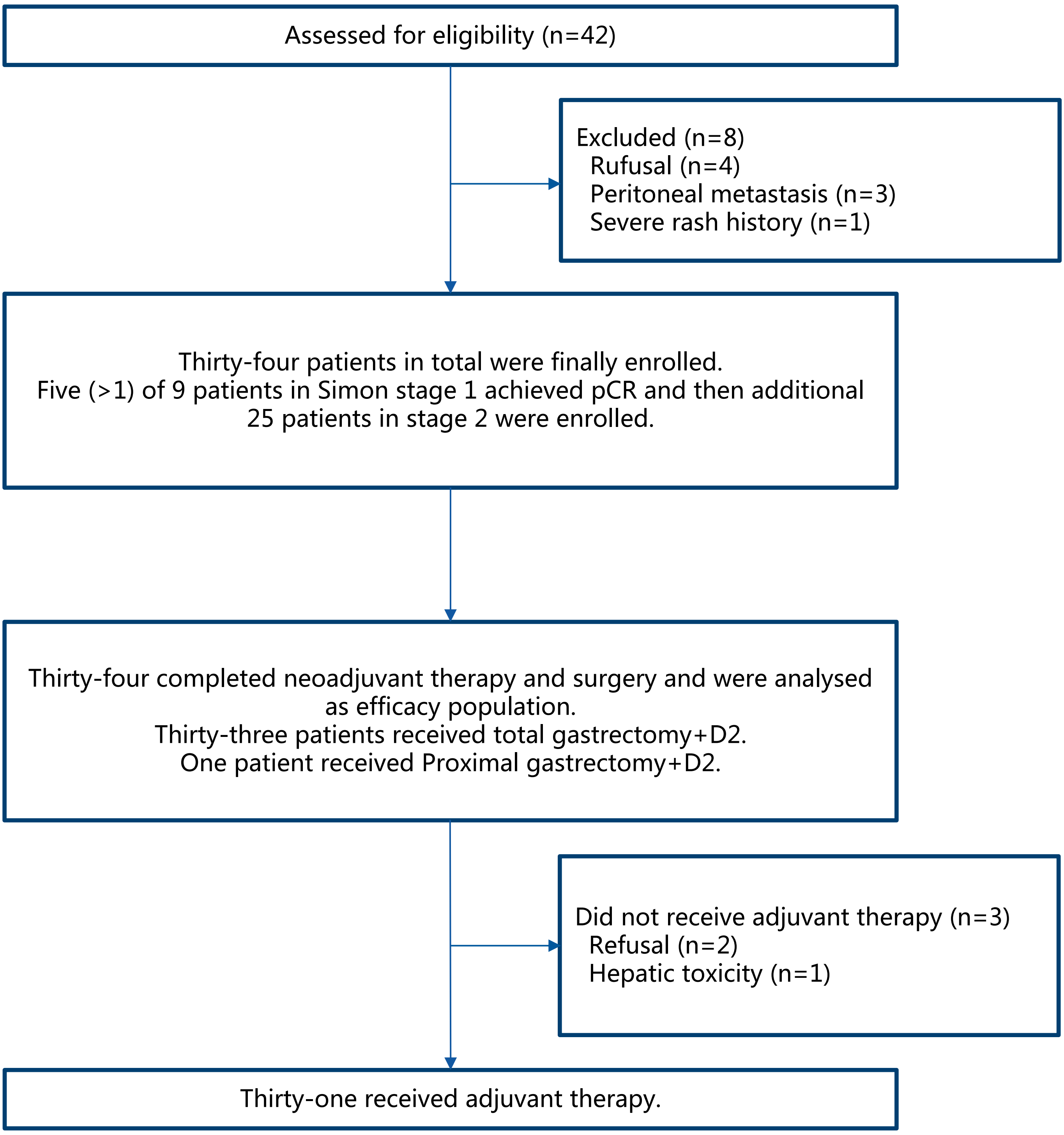


**Supplementary Figure 1. Screening and treatment flowchart of study.** pCR, pathological complete response.

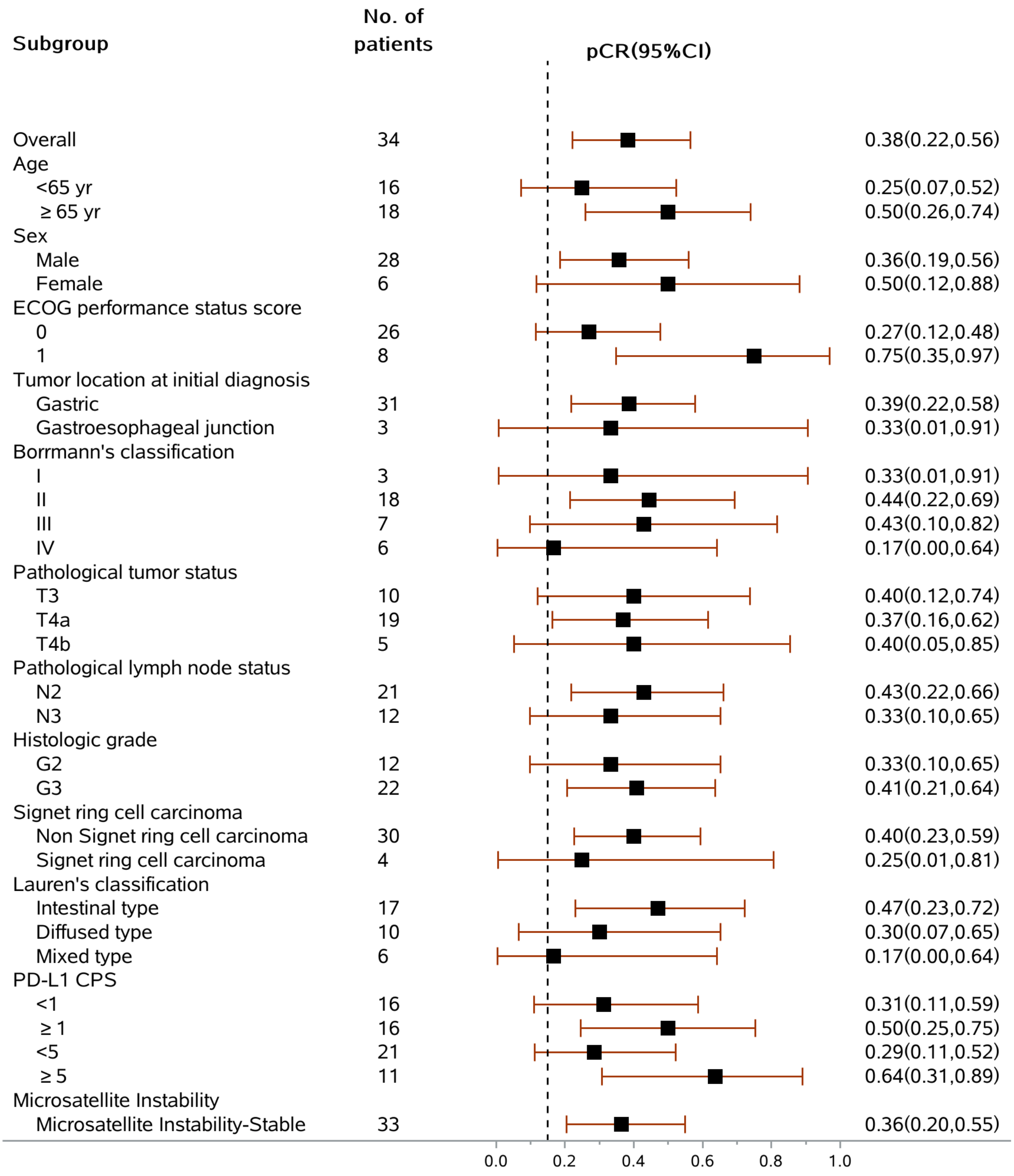


**Supplementary Figure 2. Subgroup analysis of pathological complete response (pCR).** CI, confidence interval; ECOG, Eastern Cooperative Oncology Group. PD-L1, programmed death 1 ligand; CPS, combined positive score. The centre of the error bar represented the pCR rate in different subgroup.

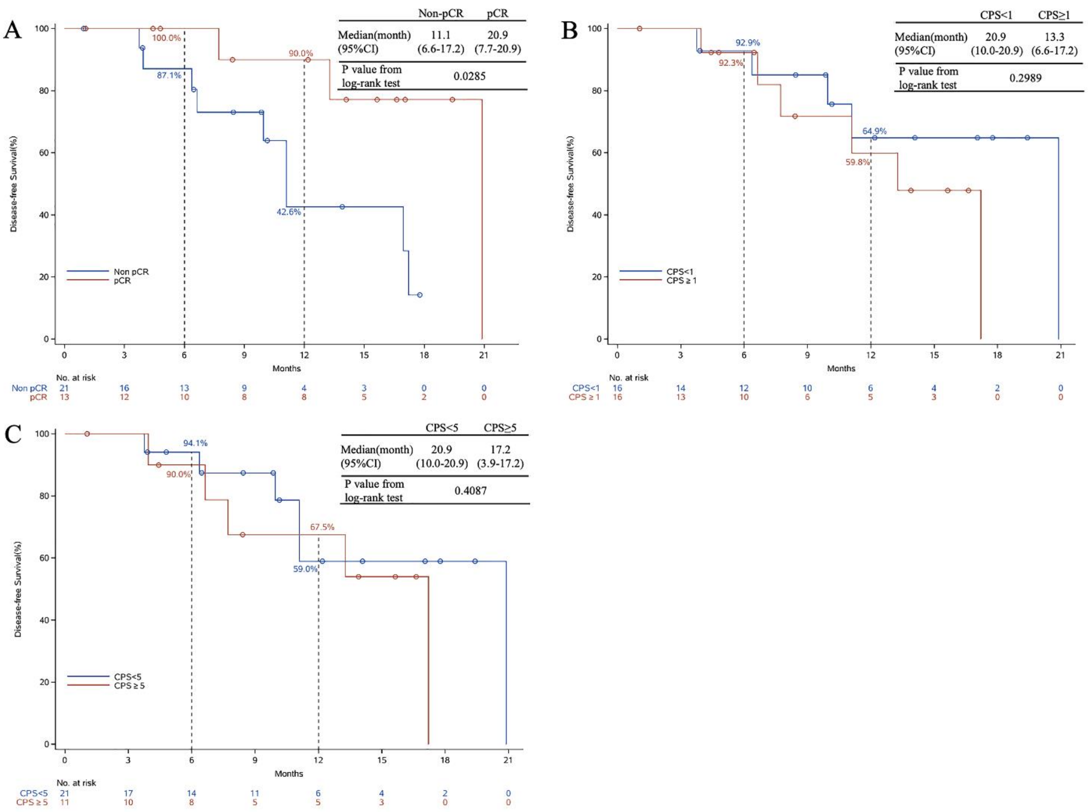


**Supplementary Figure 3. Subgroup analysis of disease-free survival.** Disease-free survival in the population stratified by pathological response (A), PD-L1 expression at a cutoff of 1 (B) and 5 CPS (C). pCR, pathological complete response; PD-L1, programmed death 1 ligand; CPS, combined positive score.

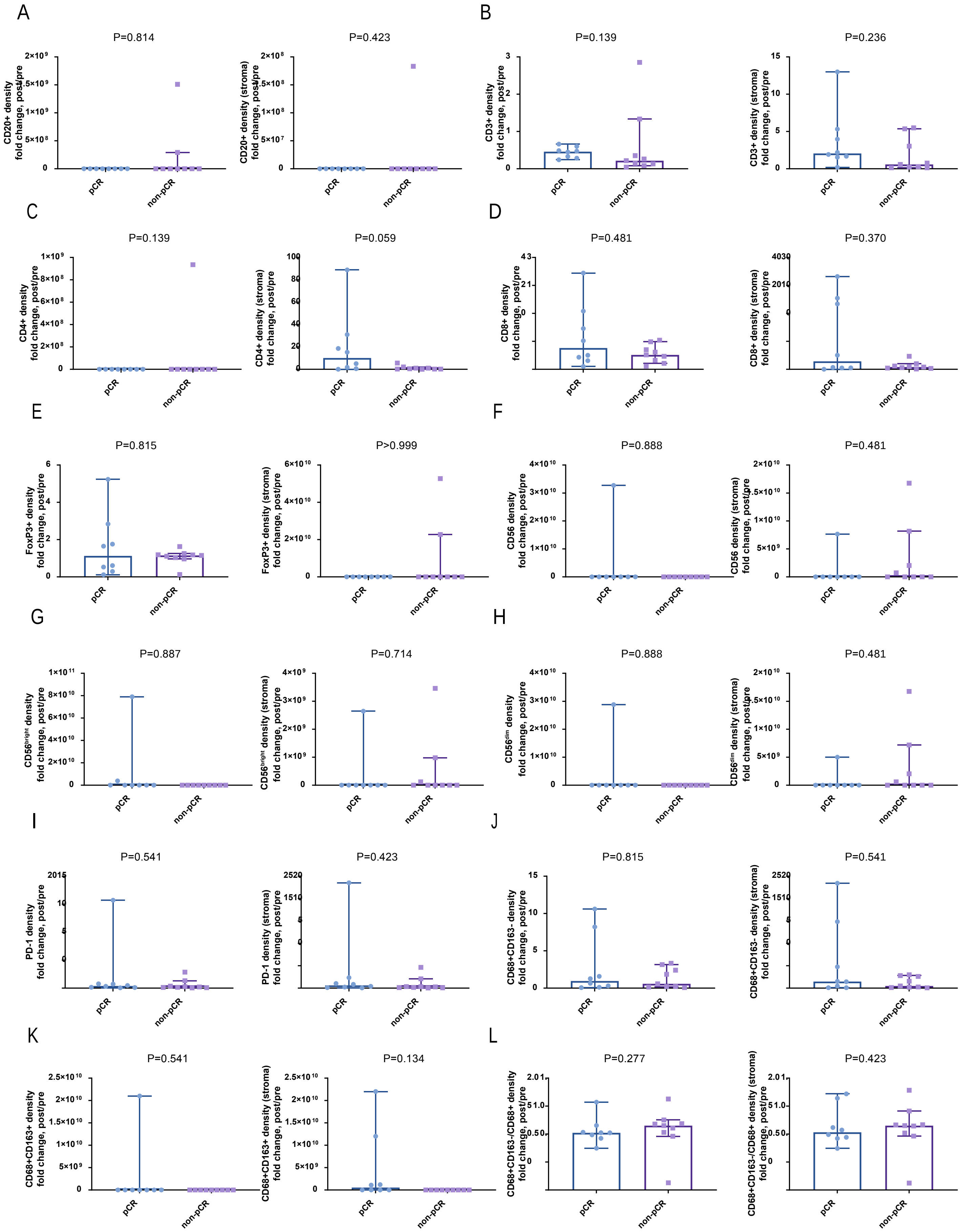

A
P=0.814
CD20+ density fold change, post/pre
P=0.423
CD20+ density (stroma) fold change, post/pre
B
P=0.139
CD3+ density fold change, post/pre
P=0.236
CD3+ density (stroma) fold change, post/pre
C
P=0.139
CD4+ density fold change, post/pre
P=0.059
CD4+ density (stroma) fold change, post/pre
D
P=0.481
CD8+ density fold change, post/pre
P=0.370
CD8+ density (stroma) fold change, post/pre
E
P=0.815
FoxP3+ density fold change, post/pre
P>0.999
FoxP3+ density (stroma) fold change, post/pre
F
P=0.888
CD56 density fold change, post/pre
P=0.481
CD56 density (stroma) fold change, post/pre
G
P=0.887
CD56bright density fold change, post/pre
P=0.714
CD56bright density (stroma) fold change, post/pre
H
P=0.888
CD56dim density fold change, post/pre
P=0.481
CD56dim density (stroma) fold change, post/pre
I
P=0.541
PD-1 density fold change, post/pre
P=0.423
PD-1 density (stroma) fold change, post/pre
J
P=0.815
CD68+CD163- density fold change, post/pre
P=0.541
CD68+CD163- density (stroma) fold change, post/pre
K
P=0.541
CD68+CD163+ density fold change, post/pre
P=0.134
CD68+CD163+ density (stroma) fold change, post/pre
L
P=0.277
CD68+CD163-/CD68+ density fold change, post/pre
P=0.423
CD68+CD163-/CD68+ density (stroma) fold change, post/pre
pCR
non-pCR

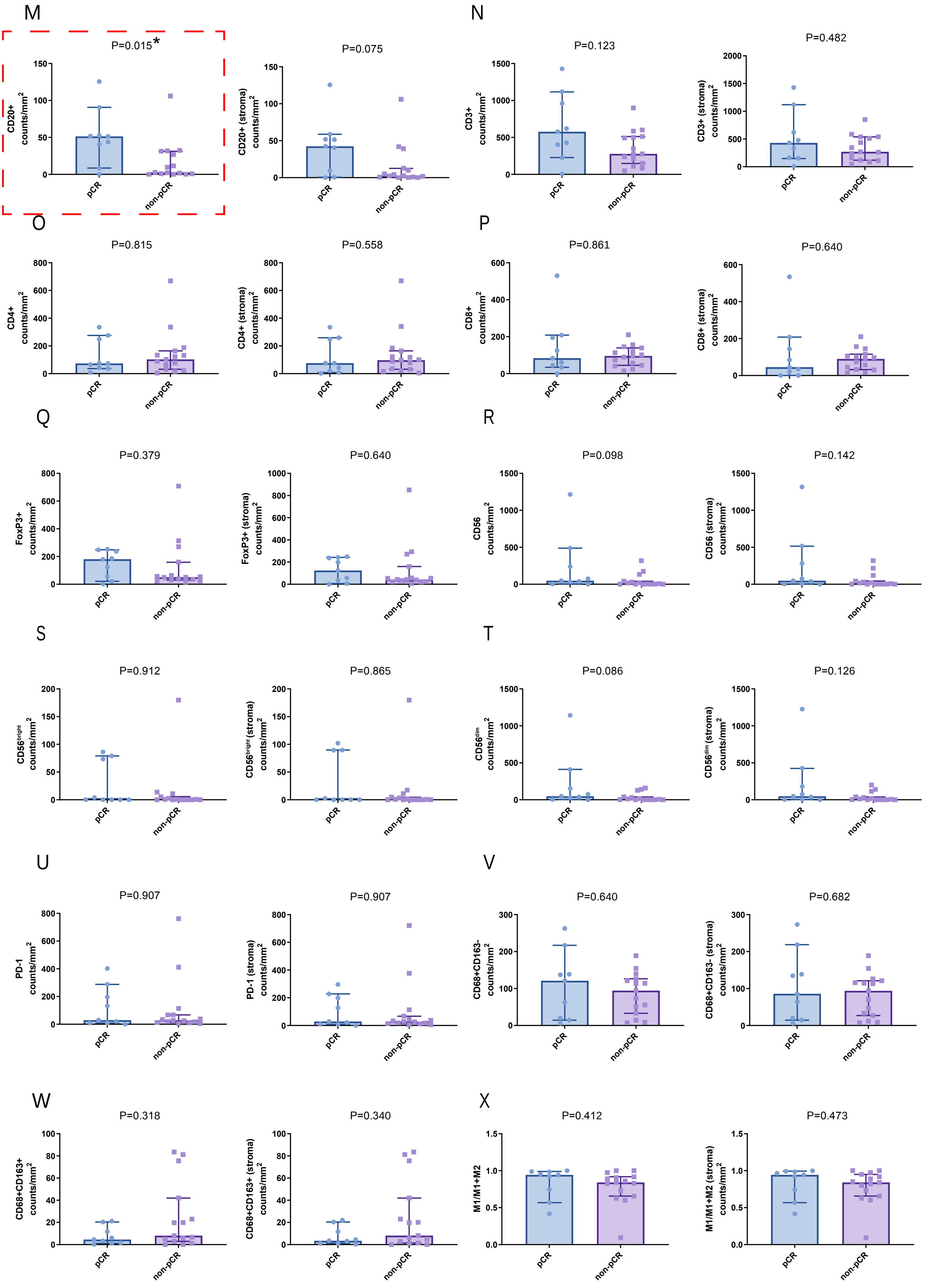

M
P=0.015*
CD20+ counts/mm2
P=0.075
CD20+ (stroma) counts/mm2
N
P=0.123
CD3+ counts/mm2
P=0.482
CD3+ (stroma) counts/mm2
O
P=0.815
CD4+ counts/mm2
P=0.558
CD4+ (stroma) counts/mm2
P
P=0.861
CD8+ counts/mm2
P=0.640
CD8+ (stroma) counts/mm2
Q
P=0.379
FoxP3+ counts/mm2
P=0.640
FoxP3+ (stroma) counts/mm2
R
P=0.098
CD56 counts/mm2
P=0.142
CD56 (stroma) counts/mm2
S
P=0.912
CD56bright counts/mm2
P=0.865
CD56bright (stroma) counts/mm2
T
P=0.086
CD56dim counts/mm2
P=0.126
CD56dim (stroma) counts/mm2
U
P=0.907
PD-1 counts/mm2
P=0.907
PD-1 (stroma) counts/mm2
V
P=0.640
CD68+CD163- counts/mm2
P=0.682
CD68+CD163- (stroma) counts/mm2
W
P=0.318
CD68+CD163+ counts/mm2
P=0.340
CD68+CD163+ (stroma) counts/mm2
X
P=0.412
M1/M1+M2
P=0.473
M1/M1+M2 (stroma)
pCR
non-pCR

**Supplementary Figure 4. Tumor immune microenvironment (TiME) of sintilimab combined with concurrent chemoradiotherapy examined by multiplex immunofluorescence (mIF).** A-L. Changes in immune cell infiltration in the tumor tissue after neoadjuvant therapy between patients achieving pCR (n=8) and those not achieving pCR (n=9). M-X. Immune cell infiltration in the tumor tissue after neoadjuvant therapy between patients achieving pCR (n=9) and those not achieving pCR (n=15). The comparison of immune cell infiltration levels was performed using Friedman's non-parametric test and the adjusted P-value using Bonferroni methods were presented in Supplementary Table 4. The error bars represented the standard deviation.

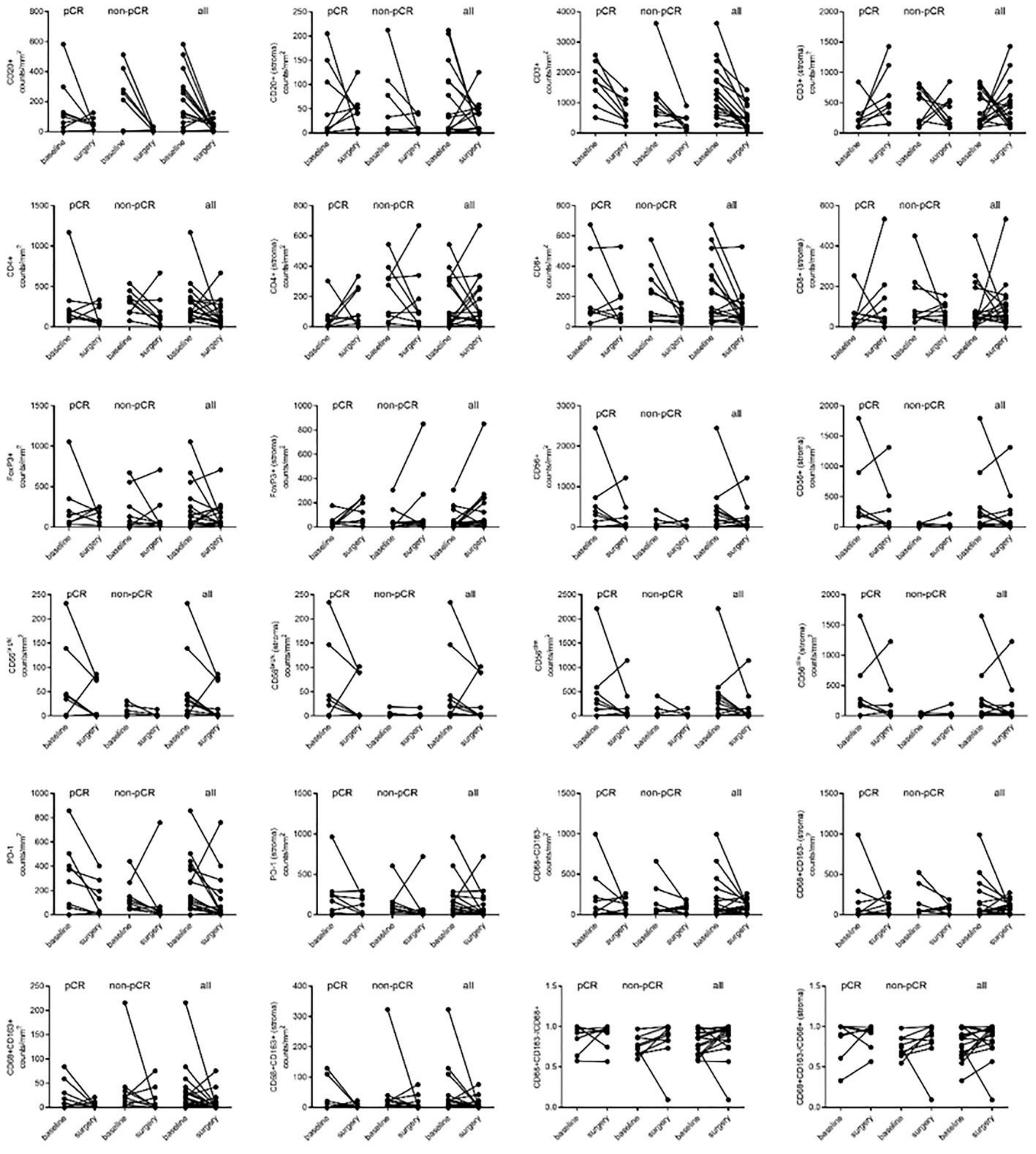


**Supplementary Figure 5. Exploratory analysis of the changes in biomarkers between patients achieving pCR (n=8) and those not achieving pCR (n=9).**

# Supplementary Note

**Perioperative Sintilimab in Combination with Concurrent Chemoradiotherapy for Locally Advanced Gastric or Gastroesophageal Junction Adenocarcinoma (SHARED): A Single-arm Phase II Trial**

**Standard Operating Procedure (SOP) for Surgery**

## 1. Anesthesia

The surgery is conducted under general anesthesia with endotracheal intubation. The decision to use epidural anesthesia as an adjunct is at the discretion of the anesthesiologist and is not specified in this protocol.

## 2. Intraoperative exploration

As per standard clinical practice, laparoscopic exploration is recommended to determine the presence of liver, peritoneal, and ovarian metastases.

## 3. Guidelines for gastrectomy:

General principles: The type of gastric resection (total or distal) should be determined by the surgeon based on tumor location, size, involvement of surrounding lymph nodes, and potential organ invasion, following the principles of radical surgery.

Margins: For tumors with expansive growth, the margin should be at least 3 cm from the tumor; for tumors with infiltrative growth, the margin should be at least 5 cm from the tumor. If these conditions cannot be met, intraoperative fast frozen pathological examination should be performed to confirm negative margins.

Lymph node dissection range: D2 lymph node dissection is performed. The lymph node dissection range for total gastrectomy includes: 1, 2, 3, 4sa, 4sb, 4d, 5, 6, 7, 8a, 9, 11p, 12a; and for distal gastrectomy includes: 1, 3, 4sb, 4d, 5, 6, 7, 8a, 9, 11p, 12a. For lesions located on the greater curvature side with a size >4cm, serosal invasion, or enlarged splenic hilum lymph nodes, lymph node dissection of the 10th group can be performed.

## 4. Guidelines for omentectomy

Whether omentectomy should be performed is not specified in this protocol.

**5. Guidelines for omental bursa excision**

Whether complete excision of the omental bursa should be performed is not specified in this protocol.

**6. Guidelines for gastrointestinal reconstruction**

The method of gastrointestinal reconstruction shall be determined by the attending surgeon based on their own experience and the specific intraoperative conditions. For total gastrectomy, esophagojejunostomy with Roux-en-Y anastomosis is recommended; for distal gastrectomy, Billroth II anastomosis with optional Braun anastomosis is recommended. Whether to use instrument-assisted anastomosis and whether to reinforce the anastomotic site with manual sutures shall be determined by the attending surgeon based on specific conditions, and is not specified in this protocol. The attending surgeon may choose laparoscopic-assisted small incision gastrointestinal reconstruction or totally laparoscopic gastrointestinal reconstruction, and is not specified in this protocol.

**7. Guidelines for surgical equipment and instruments**

The choice of energy devices, methods for vascular ligation, gastrointestinal cutting and closure devices, and instruments for gastrointestinal reconstruction shall be determined by the attending surgeon based on their experience and actual needs, and are not specified in this protocol.

**8. Guidelines for gastric tube and abdominal drainage tube**

The decision to retain a gastric tube or abdominal drainage tube postoperatively shall be at the discretion of the attending surgeon based on their experience and clinical judgment, and is not specified in this protocol.

**9. Guidelines for imaging or photographic records**

High-resolution cameras or smartphones with high-definition cameras may be used for photography, and video screenshots may be used for laparoscopic procedures. The photo

requirements are as follows:

(1) Lymph node dissection field (5 photos):

a) Left gastroepiploic vessel transection site (1 photo), must include the transection site of the left gastroepiploic artery and vein;

b) Pyloric antrum region (1 photo), must include the transection site of the right gastroepiploic artery and vein;

c) Right side of the upper edge of the pancreas region (1 photo), must include the anterior superior aspect of the hepatic artery, the anterior aspect of the lower half of the proper hepatic artery, the transection site of the right gastric artery, and the left wall of the portal vein;

d) Left side of the upper edge of the pancreas region (1 photo), must include the transection site of the left gastric artery and vein, the celiac trunk, the proximal splenic artery, and the distal splenic artery;

e) Esophageal hiatus region (1 photo), must include the crura of the diaphragm on both sides and the esophagojejunostomy site.

(2) Skin incision after closure (1 photo) (with a ruler as a reference).

(3) Fresh postoperative specimens (4 photos) (with a ruler as a reference):

a) Specimen before dissection (1 photo).

b) Specimen after dissection (3 photos): After the specimen is opened along the greater curvature of the stomach, place a ruler as a reference and take photos to record the distance from the tumor margin to the proximal resection margin (1 photo), the distance from the tumor margin to the distal resection margin (1 photo), and the size and appearance of the lesion on the mucosal surface after the specimen is spread out (1 photo).

## 10. Privacy protection and naming regulations for photos or images

All image data must be kept confidential to protect patients' personal privacy. When photos or images are used for viewing or review, personal information must be blurred or covered. The photo or image sites should be labeled with standardized Chinese names, including left gastroepiploic vessel transection site, pyloric antrum region, right side of the upper edge of the pancreas region, left side of the upper edge of the pancreas region, esophageal hiatus region, incision appearance, and specimen observation.

## 11. Criteria for confirming surgical quality

To assess the rationality of surgical procedure selection, the quality of lymph node dissection, (assistant) incision length, and specimen integrity, photo documentation will be used as previously mentioned. Full-length video recording is required for laparoscopic surgeries, and unedited video files should be stored. There is no requirement for video recording of open surgeries.

## 12. Storage of image data

All photo data should be uploaded to the data center for unified storage within 1 week after surgery, and independent backups by participating centers are allowed. Video recordings of laparoscopic surgeries should be stored and archived by each participating center.

If it is confirmed that complete photos cannot be provided according to the "Guidelines for imaging or photographic records", the case will be considered as having inadequate surgical quality and will be documented by the research committee, but the case will still be included in the study data.

## 13. Regulations for laparoscopic surgery

The brands of laparoscopic system, pneumoperitoneum support system, energy devices, trocars, and image storage devices are not specified in this protocol.

14. Intraoperative observation parameters

To be completed by the research assistant on the day of surgery. Specific parameters include:

(1) Name of the attending surgeon;

(2) Start time of surgery (in minutes), end time of surgery (in minutes), and time of specimen removal;

(3) Surgical procedure, extent of gastric resection, scope of lymph node dissection, and reconstruction method;

(4) Incision length (in centimeters);

(5) Whether there was conversion to open surgery and the reason (for laparoscopic surgery);

(6) Estimated blood loss during surgery (in milliliters; from skin incision to skin closure);

(7) Blood transfusion volume (in milliliters): In this study, blood transfusion events are defined as the administration of red blood cell suspension (ml) or plasma (ml);

(8) Tumor location (upper/middle or lesser/greater curvature, anterior/posterior wall, circumferential or not; if involving multiple areas, record according to the location of the main tumor);

(9) Maximum and minimum diameter of the tumor (in millimeters);

(10) Depth of tumor infiltration into gastric wall, presence of distant metastasis (site);

(11) Length of proximal and distal resection margins (in millimeters), surgical radicality (R0/R1/R2);

(12) Intraoperative complications (occurred from skin incision to skin closure), including:

a) Surgery-related complications: intraoperative bleeding (≥400ml) (including additional blood loss due to vascular injury), important organ injury (including gastrointestinal injury, liver injury, bile duct injury, pancreatic injury, splenic injury, and injury to other important organs and structures);

b) Pneumoperitoneum-related complications: hypercapnia, mediastinal emphysema, subcutaneous emphysema, air embolism, respiratory and circulatory instability caused by pneumoperitoneum pressure;

c) Anesthesia-related complications.

(13) Intraoperative death (from skin incision to skin closure), regardless of the cause.

# Radiotherapy Protocol of Perioperative Sintilimab in Combination with Concurrent Chemoradiotherapy for Locally Advanced Gastric or Gastroesophageal Junction Adenocarcinoma (SHARED) Version 1.0

## 1 Selection of radiotherapy technology

Conformal, intensity-modulated, or spiral tomographic intensity-modulated techniques can be employed for gastric cancer radiotherapy. Generally, 6-8 MV X-rays are used for conformal radiotherapy, with four to five shooting fields. X-ray at a dose of 6 MV is recommended for intensity adjustment of the fixed field. Generally, five to seven fields should be set up, and both shoulders should be avoided as far as possible. In general, X-ray at a dose of 6 MV and 2-arc isocentric coplanar irradiation are used in the spiral tomographic intensity-modulated technique. It is not recommended to use dosing mode of simultaneous integrated boost (SIB), i.e., increasing the radiation dose to gastric tumors without increasing the radiation dose to normal tissues.

## 2 Positioning and simulation

Before the simulation, patients will be instructed to fast for at least 3 h and drink 300 ml fluid diet 10 min before CT simulation and daily radiotherapy to maintain a consistent stomach volume.

The patient is asked to assume the supine position and hold his or her elbows up to his or her forehead, and this position is fixed with thermoplastic body film or a vacuum pad/foam. Oral contrast agent or intravenous angiography can help to localize the target area during CT scan.

CT simulation: The scanning condition can be set as axial scanning with a layer thickness of 3 mm, and the scanning range can be set according to the lesion location and typical range should include thoracic and abdominal cavity. To manage respiratory movement, CT scanning can be combined with technologies such abdominal compression, four-dimensional CT, and respiratory gating.

## 3 Definition of the targeted area

### 3.1 Gross tumor volume (GTV)

This includes primary tumors (GTVp) and metastatic lymph nodes (GTVnd). GTVp is a visible gastric or gastroesophageal junction lesion that can be determined using a combination of imaging techniques (e.g., esophagography, contrast-enhanced CT, MRI, and/or PET/CT) and endoscopy. GTVnd refers to metastatic lymph nodes with a diameter of ⩾10 mm as observed on CT and/or MRI or a high SUV (except inflammatory lymph nodes) as observed on PET/CT. Even if the lymph node characteristics are under these standards, those with evident necrosis, circular enhancement, enhancement to a similar degree as that of the primary lesion, and eccentric calcification are also considered as GTVnd.

**3.2 Clinical target volume (CTV)**

Based on the location of the primary tumor and the degree of invasion, lymph node metastasis, CTV includes GTV, GTVnd and the high-risk lymphatic drainage area. According to the National Comprehensive Cancer Network (NCCN) guidelines and uropean Organization for Research and Treatment of Cancer (EORTC) contouring guidelines for neoadjuvant RT for gastric cancer, **selective lymph node irradiation** is recommended for radical radiotherapy. For selective lymph node irradiation, in addition to the primary lesion and metastatic lymph node area, the corresponding lymph node drainage area with a high lymph node metastasis rate should also be included. The following is for reference (EORTC standard):

| Tumor location | Lymphatic drainage area requiring radiotherapy |
|---|---|
| Siewert I type GEJ | No.7,9,11p,19,20,110–112 |
| Siewert III type GEJ | No.7,9,10,11p,11b,19,20,110,111 |
| Upper 1/3 GC | No.7,9,10,11p,11b,19 |
| Middle 1/3 GC | No.7,8a,8p,9,10,11p,11b,18,19 |
| Lower 1/3 GC | No.7,8a,8p,9,10,11p,12,13,17,18 |

Given the adverse effects of combination therapy, the inclusion of the whole stomach into CTV should be avoided. If the selective lymph node irradiation area does not meet the above requirements, **involved field irradiation** may be considered. For involved field irradiation, CTV is defined as GTVp with a 30-mm expansion superiorly and inferiorly and a 5-mm expansion in other directions and GTVnd with a 5-mm expansion in all directions (adjusted when the anatomical barrier is included).

**3.3 Planned target area (PTV)**

This includes CTV with a 5-mm expansion in all directions; longitudinal expansion can be up to 8 mm, and the actual margin can be determined according to the quality control data of each radiotherapy system.

### 3.4 Organs at risk (OAR)

This mainly includes the spinal cord, lungs, heart, liver, trachea, main bronchus, stomach， small intestine， colon, and kidneys.

## 4 Radiotherapy dose and regimen

The 45 Gy dose of radiotherapy will be delivered in 25 fractions within 5 weeks typically.

## 5 Normal Structures and Constraints

The dose limits according to QUANTEC (2012) are as follows:
The maximum doses for cervical and thoracic spinal cords are ≤45 Gy and ≤50 Gy, V20 is ≤30%.The mean cardiac dose is <26 Gy. The mean liver dose is <30 Gy, or <28 Gy in patients with previous liver diseases or hepatocellular carcinoma with Child－Pugh class A liver function.
It is recommended that the stomach volume of 40 Gy should be <50% of the entire stomach and Dmax stomach≤50 Gy.
Other dose limitations of the organs at risk (OARs) are shown as below: V20 kidney<26%, Dmean kidney<18Gy, V10 bone marrow<26%, Dmax intestine≤42 Gy, and V40 intestine<10%.

## 6 Image-guided technology

Pre-radiotherapy image guidance for gastric or gastroesophageal junction cancer includes two- and three-dimensional online images. The online images should be collected before the first week treatments and then once a week. Megavoltage CT (MVCT) scans will be performed daily when treatment regimen delivered by helical tomotherapy (TOMO) system.

## 7 Quality assurance

The quality assurance (QA) team associated with this study will include experienced experts in the field of radiotherapy, dosimetry, medical physics, medicine, surgery, imaging, pathology, and data censors. Diagnostic images before and after

neoadjuvant treatment will be reviewed centrally and independently by two radiologists. The first three cases of target volume delineation will be reviewed centrally and then checked randomly. This QA process includes: (1) a data integrity review for completeness of protocol-required elements, the format of data, and possible data corruption, and recalculation of dose-volume histo grams, (2) a review of compliance with target volume and organ-at-risk contours by study chairs and (3) a review of dose prescription and dose heterogeneity compliance. Data censors in this study will stay in communication with researchers and check the quality of the data collection randomly.
